# Moving Backgrounds Confer Age-Related Positional Uncertainty on Flash-Grab Targets

**DOI:** 10.1177/2041669519879178

**Published:** 2019-09-25

**Authors:** Stuart Anstis

**Affiliations:** Department of Psychology, UC San Diego, La Jolla, CA, USA

**Keywords:** motion, flash-lag, flash-grab, illusions, aging, uncertainty

## Abstract

The flash-grab effect made a stationary flashing cross appear to jump back and forth through a distance of more than 2°. Observers were asked to move a cursor as quickly as possible on to this flashing target. All observers younger than 65 years, and 39% of those over 65 years, could do this without difficulty within 1 second to 2 seconds. But 61% of those over 65 years experienced uncertainty about the exact position of the target and took from 6 to 147 seconds to hit it—about 4 times longer than to hit an actually jumping cross. This loss of hand–eye coordination was probably perceptual, not motor.

The perceived positions of stationary objects can be shifted by nearby motion (Whitney, 2002; Whitney & Cavanagh, 2000). These effects include a family of flash-lag effects, in which a flash and a moving object that are exposed in the same location are perceived to be displaced from one another (Eagleman & Sejnowski, 2000, 2007; Nijhawan & Khurana, 2010). Specifically, Cavanagh and Anstis (2013) and Anstis and Cavanagh (2017) have discovered a flash-grab effect, in which moving backgrounds massively change the apparent size, shape, and orientation of flashed test objects.

Movie 1. shows an example of a flash-grab illusion. A cross flashes in place and is alternately red and blue. The moving background consists of a 2.8° square made of dashed lines that follows a counterclockwise square trajectory, moving left, then straight down, then right, and then straight up. The square trajectory is the same size as the square itself. The red and blue crosses are in exactly the *same* location, but they are flashed at the moment when opposite corners of the moving square pass through that location. The red (blue) cross flashes when the top left (bottom right) corner of the moving square passes through the location of the cross. As a result, the red and blue crosses appear to lie at the top left and bottom right corners of the square, respectively. As in all flash-grab stimuli, each flashed target appears to be dragged along the direction of the background motion that follows (not precedes) the flash.

**Movie 1 (Click to play).** The flash grab illusion. The red and blue crosses are in the same place, but appear displaced in the direction in which the square moves after each flash.

Eighteen observers (O’s), whose ages ranged from 19 to 82 years, used a matching method to measure the perceived extent of the flash-grab illusion (*x*-axis in Figure 2). Free eye movements were permitted in all experiments. Two steadily illuminated crosses, one red and one blue, were positioned 8° away from the flash-grab stimulus, and O used the cursor to adjust the separation between them to match the perceived gap between the flash-grab red and blue crosses (the actual gap between the two flashed crosses was zero, since these two crosses flashed in alternation at exactly the same location). The diagonal of the moving square was 4° of visual angle, and O’s settings varied from 5% to 172% of that, with a median value of 61% of 4° (=2.4°)—far larger than most flash-lag illusions. Most O’s refused to believe that the red and blue flashing crosses were actually congruent and were convinced that the cross was jumping back and forth.

## Positional Uncertainty

**Figure 1.**
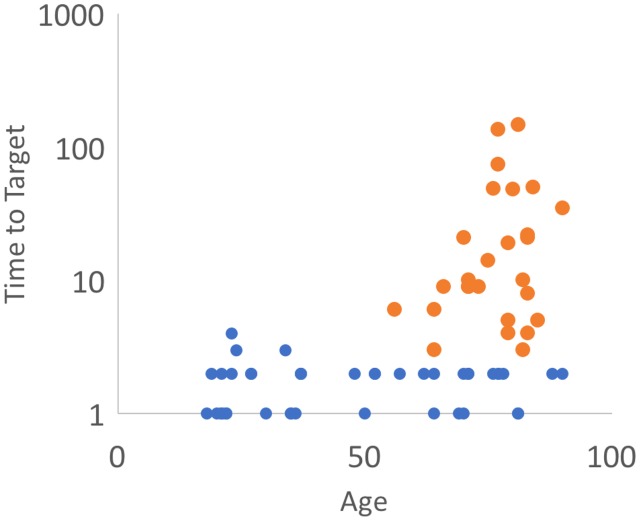
Time-to-target (s) versus age. Note that 61% of all seniors over 65 years were slow to hit the target (yellow datum points).

We now show that the moving background shifted both the mean and variance of the perceived positions of the crosses. Seventy-three healthy observers (including the previous eighteen) were tested one at a time on a simple hand or eye coordination task. Their ages ranged from 18 to 93; 32 of them were younger than 65 and 41 years were older. O’s used a trackpad to move the cursor from a corner of the screen until it hit a stationary target spot centered on the screen. This was a trivially easy task, taking less than 2 seconds. But many seniors were substantially slower at hitting the flash-grab target (Figure 1).

We measured the time it took each observer to hit the flashing target. O’s younger than 65 years could hit the flashing target just as quickly as the stationary target. This was also true for 39% (16 of 41) of the O’s aged 65 years or older. But for the other 61% (25 of 41) the time-to-target increased substantially, and their times ranged from 6 to 147 seconds (median time was 9.5 seconds, mean time was 26.4 seconds). Observers typically moved the cursor rapidly to the immediate vicinity of the target, but then hunted around the target, having difficulty in exactly hitting it, often expressing their frustration.

**Figure 2.**
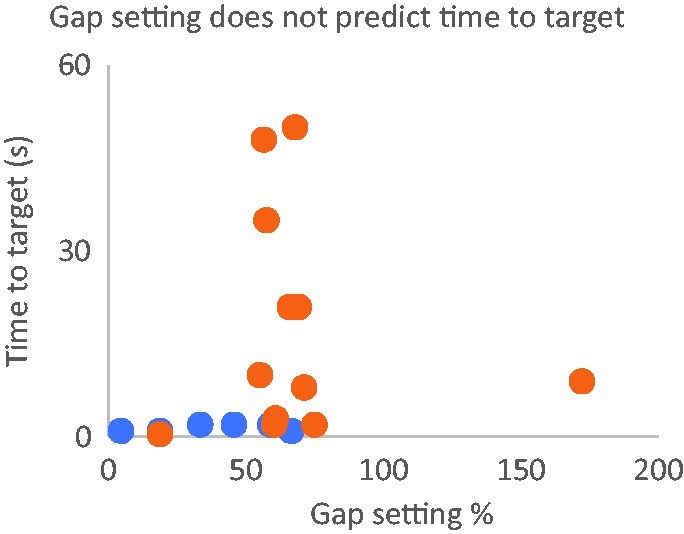
*x* = Perceived extent of the flash-grab illusion, expressed as percentage of the moving square’s diagonal, for 11 observers aged <65 years (blue points) and 7 observers aged >65 years (yellow points). (*x*) did not significantly affect the time to target (*y*).

We wondered whether the slowest individuals were those who made the largest gap setting and so presumably perceived the greatest illusory movement. However, we found little correlation (*R* = .217) between the matched gap setting and the time-to-target (Figure 2).

**Figure 3.**
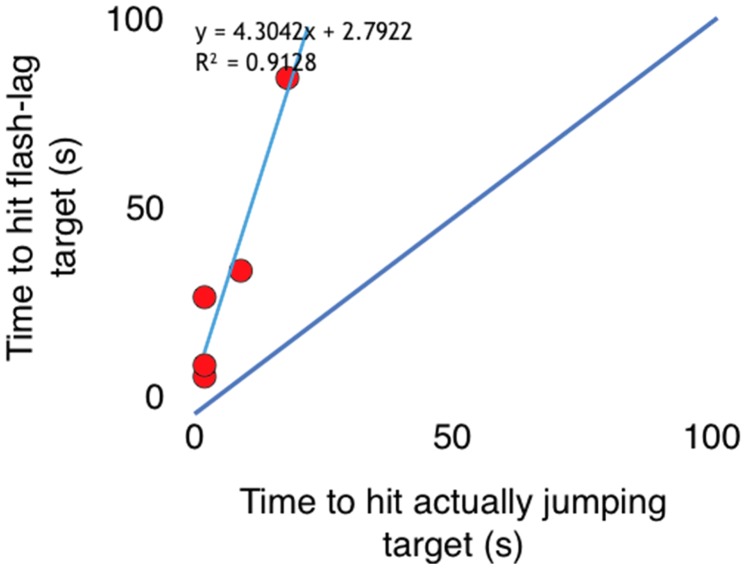
Five slow seniors took 3 to 13 times longer to hit the stationary flash-grab cross (*y*) than the actually jumping cross (*x*). Thus, *y *≫* x*.

Five slow seniors from Figure 1, aged 72 to 85 years, viewed three trials each of (a) a stationary red or blue flash-grab cross that only appeared to move, as in Figure 1, and (b) a cross that actually jumped back and forth in apparent motion between two positions 4° apart (but with no moving square). Figure 3 shows that paradoxically, (a) proved far harder to hit than (b). We speculate that a cross that appeared to move but did not had greater positional uncertainty than a cross that actually moved, making it a much more elusive target.

In short, the slow seniors were not hampered by motor difficulties, since from a starting position 8° away they could usually hit a stationary control target in <2 seconds in pretests, and they could even hit a target cross faster when its displacement was real rather than illusory. The flash-grab excursions looked equally large to slow and fast seniors (Figure 2). The slowing-down that affected 61% of our seniors is still a mystery, but this flash-grab task may conceivably become a diagnostic one day for a so far unknown age-related loss of capacity.
